# Causes of brain dysfunction in acute coma: a cohort study of 1027 patients in the emergency department

**DOI:** 10.1186/s13049-019-0669-4

**Published:** 2019-11-07

**Authors:** Wolf Ulrich Schmidt, Christoph J. Ploner, Maximilian Lutz, Martin Möckel, Tobias Lindner, Mischa Braun

**Affiliations:** 1 Department of Neurology, Charité – Universitätsmedizin Berlin, Freie Universität Berlin, Humboldt-Universität zu Berlin and Berlin Institute of Health, Augustenburger Platz 1, 13353 Berlin, Germany; 2 Center for Stroke Research, Charité – Universitätsmedizin Berlin, Freie Universität Berlin, Humboldt-Universität zu Berlin and Berlin Institute of Health, Berlin, Germany; 3 Department of Emergency Medicine, Charité – Universitätsmedizin Berlin, Freie Universität Berlin, Humboldt-Universität zu Berlin and Berlin Institute of Health, Berlin, Germany

## Abstract

**Background:**

Coma of unknown etiology (CUE) is a major challenge in emergency medicine. CUE is caused by a wide variety of pathologies that require immediate and targeted treatment. However, there is little empirical data guiding rational and efficient management of CUE. We present a detailed investigation on the causes of CUE in patients presenting to the ED of a university hospital.

**Methods:**

One thousand twenty-seven consecutive ED patients with CUE were enrolled. Applying a retrospective observational study design, we analyzed all clinical, laboratory and imaging findings resulting from a standardized emergency work-up of each patient. Following a predefined protocol, we identified main and accessory coma-explaining pathologies and related these with (i.a.) GCS and in-hospital mortality.

**Results:**

On admission, 854 of the 1027 patients presented with persistent CUE. Their main diagnoses were classified into acute primary brain lesions (39%), primary brain pathologies without acute lesions (25%) and pathologies that affected the brain secondarily (36%). In-hospital mortality associated with persistent CUE amounted to 25%. 33% of patients with persistent CUE presented with more than one coma-explaining pathology. In 173 of the 1027 patients, CUE had already resolved on admission. However, these patients showed a spectrum of main diagnoses similar to persistent CUE and a significant in-hospital mortality of 5%.

**Conclusion:**

The data from our cohort show that the spectrum of conditions underlying CUE is broad and may include a surprisingly high number of coincidences of multiple coma-explaining pathologies. This finding has not been reported so far. Thus, significant pathologies may be masked by initial findings and only appear at the end of the diagnostic work-up. Furthermore, even transient CUE showed a significant mortality, thus rendering GCS cutoffs for selection of high- and low-risk patients questionable. Taken together, our data advocate for a standardized diagnostic work-up that should be triggered by the emergency symptom CUE and not by any suspected diagnosis. This standardized routine should always be completed - even when initial coma-explaining diagnoses may seem evident.

## Introduction

Coma of unknown etiology (CUE) denotes an acute impairment of consciousness that is not caused by traumatic brain injury (TBI) or cerebral hypoperfusion due to cardiac arrest. The underlying pathologies can broadly be classified into primary intracranial disorders that focally affect the ascending reticular activation system and primarily extracranial disorders that diffusely affect brain structures [1]. Both categories include highly time-sensitive medical, neurological and neurosurgical emergencies [2]. A fast but comprehensive diagnostic work-up is mandatory, since in many cases good outcome depends on early treatment, e.g. in meningo-encephalitis [3] or basilar artery occlusion [4]. Current emergency medicine guidelines do not yet include a generally accepted algorithm for initial management of CUE. In order to develop standard operating procedures and to define the essentials of an efficient diagnostic work-up, detailed knowledge of the spectrum of pathologies in CUE patients is a prerequisite. Clinicians are aware of this problem and reviews and educational articles have been published on this question [2, 5–7]. However, since their hallmark description of 500 non-traumatic coma patients in an intensive care unit (ICU) by Plum and Posner in the 1970’s [1], only a small number of observational studies on comatose patients from ICUs and EDs have been published [8, 9]. In these studies, the prevalence of focal vs. diffuse pathologies varies from 28 to 64% vs. 37–75% [8]. In a large cohort recruited from a medical ICU [10], only 9% suffered from cerebrovascular events or CNS infections. By contrast, the prevalence of structural brain damage was 36–60% when neurology or neurosurgery departments were included [11–14]. Moreover, while intoxication is a major cause of CUE in one ED cohort [15], other studies excluded intoxicated patients [11, 13].

One factor contributing to the heterogeneity of data is the way pathologies are classified. For example, coma following epileptic seizures may be classified as a diffuse brain disorder but may also relate to an acute focal lesion of the CNS that does not directly affect the ascending reticular activation system. However, as this information is rarely available during initial management, a more useful classifier for these cases may be the presence or absence of acute focal CNS damage. Therefore, an additional class of pathologies may be introduced that includes primary brain disorders without acute focal damage, such as epilepsy syndromes (as opposed to acute symptomatic epileptic seizures), and coma mimics such as psychiatric disorders (e.g. akinetic mutism or dissociative state) and neuromuscular paralysis in neurodegenerative disorders. In addition, there is only limited data about patients presenting with more than one cause of coma [13]. CUE patients may present with multiple pathologies that might each be a self-contained explanation for coma (e.g. intoxication and intracranial hemorrhage or cerebral infarction and hypoglycemia). In retrospect, such pathologies may well turn out to be pathophysiologically coherent, but this may not necessarily be evident during initial management in the ED. Thus, diagnostic work-up risks to be prematurely terminated as soon as the first coma-explaining pathology is detected.

Of course, experienced ED physicians are aware of the different causes of coma. However, there is a substantial lack of data on their exact frequencies of occurrence in a real-life patient population. And besides, there is even less empirical insight into the fact that multiple coma-explaining pathologies may appear simultaneously in one patient. We aim to provide clear-cut data on the spectrum of pathologies underlying CUE to form the basis for a rational emergency management that tailors the available resources to the specific requirements of these patients.

## Methods

### Setting

Charité Campus Virchow-Klinikum is a tertiary care university hospital located in central Berlin, Germany, caring for approximately 70,000 ED patients per year [16].

### Patients/study size

Between May 2013 and January 2017, we identified *n* = 1027 consecutive ED patients presenting with CUE. Comatose patients with primary cardiac arrest or TBI were not included. All patients were examined by fully trained paramedics, emergency physicians and neurologists. The level of consciousness was determined by assessing the ability to communicate verbally or by eye, head or limb movements [17] and by repeated Glasgow Coma Scale (GCS) evaluation before and on admission to the ED. Since there is significant inter-observer variability in GCS assessment [18] and CUE may fluctuate before and during initial work-up we deliberately did not apply GCS cutoffs for definition of coma. All patients received a defined set of emergency diagnostics as described previously [19], with clinical examination (including full neurological examination), ECG, blood gas analysis, laboratory tests including toxicology, cranial imaging and cerebro-spinal fluid (CSF) testing.

### Categorization of pathologies

In order to determine individual causes of CUE, the authors reviewed each patient’s clinical, laboratory, radiology (including follow up CT or MRI scans) and autopsy findings (if available). One main diagnosis was allocated to each patient. Main diagnoses were categorized into three main classes: I) primary central nervous system (CNS) pathology with proof of acute structural or inflammatory brain damage on imaging or CSF testing, II) primary CNS pathology without proof of acute structural brain damage, III) acute secondary CNS disorders. Patients were grouped into class I whenever an acute structural brain damage was present that could explain coma either directly (by its specific localization and/or size) or indirectly (e.g. by acute symptomatic seizures or subsequent metabolic disturbances). Patients with status epilepticus or post-ictal state were grouped into class II when there was no proof of acute structural brain damage or other triggers for acute symptomatic seizures and when patients could be (or had been previously) diagnosed with epilepsy. Accordingly, class II included other primary CNS pathologies without acute structural brain damage such as neuro-degenerative disease or psychiatric disorders. Class III included patients with primary extracranial disorders that affected the CNS secondarily such as acute metabolic disturbances, intoxication and surgical emergencies (e.g. aortic dissection or mesenteric ischemia).

In addition to the main diagnosis, we determined all further pathologies detected during each patient’s ED management that would have been self-contained explanations for CUE (e.g. respiratory insufficiency/CO_2_ narcosis with subsequent proof of opiate intoxication or ethanol intoxication with subsequent proof of subdural hematoma). In cases of multiple and/or intertwined medical disorders, these were ranked according to severity, causality and timeline in order to define main diagnosis and accessory pathologies.

### Diagnostic criteria

For diagnoses supported by laboratory results such as respiratory and metabolic causes of coma, we applied laboratory cut-off values. Given that the patient’s past medical history is often unknown or incomplete in the ED, we applied the following values based on a healthy (non-adapted) individual: hypercapnia, pCO_2_ > 65 mmHg; hypoglycemia, serum glucose < 2.2 mmol/l (< 40 mg/dl); hyperglycemia, serum glucose > 25 mmol/l (> 450 mg/dl); hyponatremia, serum Na^+^ < 115 mmol/l; hypernatremia, serum Na^+^ > 160 mmol/l (according to [20]). Detection of hepatic or uremic encephalopathy was based on clinical criteria supported by laboratory constellations according to Vilstrup et al. [21] and Seifter et al. [22]. Septic encephalopathy required positive systemic inflammatory response syndrome criteria followed by proof of septiceamia [23]. Ethanol intoxication was based on serum alcohol levels > 17.4 mmol/l and considering the patient’s age, sex and past medical history. Intoxication with other substances was ascertained when a) toxicological tests were positive and/or b) antagonizing resulted in a markedly increased alertness and/or c) the patient or reliable third parties could retrospectively provide a conclusive history of ingestion of (illegal) drugs. A hypothermia-induced coma was asserted when core body temperature was less than 32 °C [24]. In order to distinguish between metabolic derangement in epilepsy and acute symptomatic seizures in metabolic disease, we used the cut-off values proposed by Beghi et al. [20]. Cardiogenic shock was defined according to Reynold et al. [25] and diagnosed based on clinical criteria. Note that primary cardiac arrest patients were not included in our cohort. Here, cardiogenic shock means (secondary) cardiac arrest or cardiogenic cerebral hypoxemia with a later onset in the wake of other pathologies (e.g. subarachnoid hemorrhage or intoxication).

### Variables

We investigated categorized main diagnoses and accessory pathologies and the quantitative and qualitative variables age, sex, GCS values, intubation on arrival in the ED (y/n) and death in hospital (y/n).

### Statistical methods

Descriptive statistics and demographics were reported as medians and interquartile ranges (IQR). Differences in the spectrum of diagnoses between groups were tested by χ^2^-test. A *p*-value below .05 was considered significant. We used IBM SPSS Statistics for Windows 22.0 (IBM, USA) for data analysis.

### Ethics approval

An ethics vote for the analysis of routinely acquired clinical data was obtained from the Ethics Committee of the Charité-Universitätsmedizin Berlin (“Emergency Processes in Clinical Structures”, EA1/172/14).

## Results

Here, we report on a large cohort of 1027 consecutive ED-patients presenting with CUE. Data were deliberately collected and analyzed from the perspective of an ED physician who is acutely confronted with a highly ambiguous emergency. We applied a modified classification that takes into account the possibility of primary CNS-disorders without focal damage and systematically investigated the possibility of multiple CUE-explaining pathologies.

In all, there were 1027 patients with CUE who arrived with ambulances to the ED between May 2013 and January 2017, resulting in a rate of 0.4% of all ED patients. In 854 of these 1027 patients, coma persisted at least until all emergency diagnostics were completed (‘persistent CUE’: 463 males, 391 females; median age 65, IQR 47–77 yrs.; median GCS 5, IQR 3–8). One hundred seventy-three of the 1027 CUE patients had spontaneously re-gained consciousness on arrival in the ED or before emergency diagnostics were completed (‘transient CUE’: 107 males, 66 females; median age 65, IQR 47-77 yrs.; median GCS [on-site] 8, IQR 4–10; median GCS [in ED] 14, IQR 11–15).

### Persistent CUE – main diagnoses (Fig. 1, Table 1)

Three hundred thirty of 854 persistent CUE patients suffered from an acute CNS pathology (class I, 39%) with either primarily supra-tentorial (*n* = 278, including inflammatory CNS disease) or infra-tentorial structural damage (*n* = 52). Diagnoses included hemorrhage (*n* = 190), infarction (*n* = 95), inflammation (*n* = 22), posterior reversible encephalopathy syndrome (*n* = 5), tumorous lesions (focal edema, exacerbated hydrocephalus or newly diagnosed solid tumor; *n* = 17) and sinus thrombosis (*n* = 1). Two hundred thirteen of 854 patients were diagnosed with primary CNS pathologies without acute structural damage (class II, 25%). These were mainly status epilepticus (*n* = 94) or post-ictal states (*n* = 94) in the context of epilepsy syndromes or following first unprovoked seizures (as opposed to acute symptomatic seizures). Rarer pathologies included psychiatric disease (*n* = 21) and neuro-degenerative disease (*n* = 4). An acute secondary CNS affection was present in 311 of 854 patients (class III, 36%). Main diagnoses included medical pathologies such as intoxication (*n* = 165), cardiac (e.g. cardiovascular failure without necessity of medical or mechanical reanimation or prolonged cardiogenic syncope) or pulmonary disease (*n* = 53), metabolic or homeostatic disturbance (*n* = 50), septic encephalopathy (*n* = 25), and prolonged non-cardiogenic syncope (*n* = 2). In six patients, CUE was related to surgical emergencies such as aortic dissection/bleeding (*n* = 5) and mesenteric infarction with severe lactic acidosis (*n* = 1). In 10 patients, the pathology causing CUE could not be identified with certainty. Nevertheless, we included these patients in class III because an acute structural CNS pathology had been ruled out by all available means and there was no indication for epileptic seizures/epilepsy or any other neurological or psychiatric disease. Intubated patients and non-intubated comatose patients showed significantly different distributions of main diagnoses (χ^2^-test: *p* < .000). In intubated patients, primary brain damage was more frequent (class I, 53% vs. 31%) while primary CNS pathologies without acute structural damage were less frequent (class II, 15% vs. 30%). Main diagnoses from class III varied between 31% (intubated) and 39% (non-intubated).

**Fig. 1:**
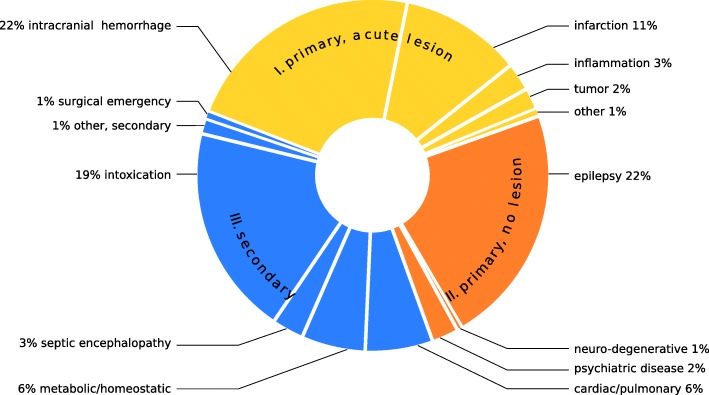
Distribution of main diagnoses in 854 patients with persistent CUE (s. Table 1), ordered by classes I-III (463 males, 391 females; median age 65; median GCS 5)

**Table 1:**
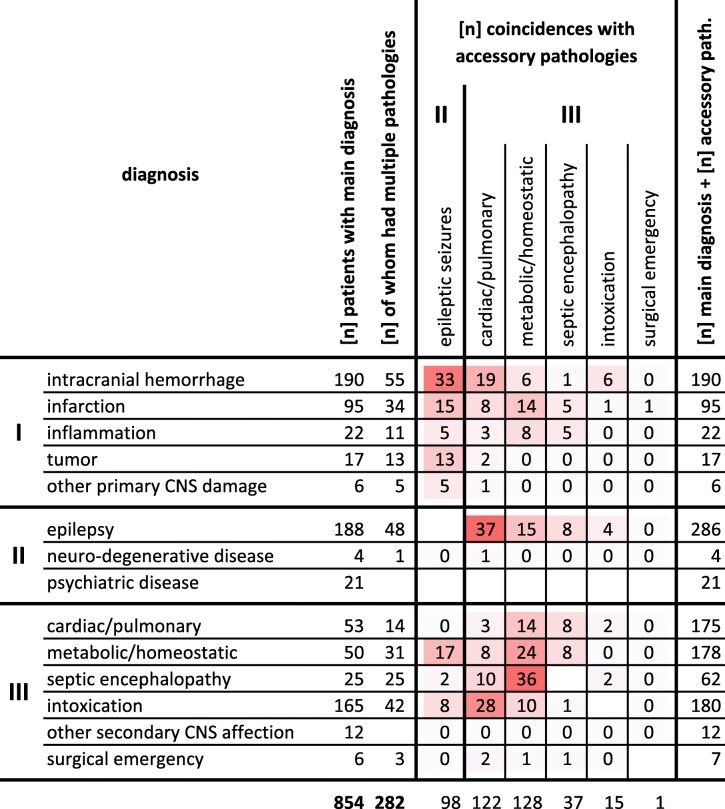
Left-hand columns – frequencies of main diagnoses in 854 patients with persistent CUE (463 males, 391 females; median age 65; median GCS 5), ordered by classes I-III. Second column of numbers – numbers of patients diagnosed with accessory pathologies besides their main diagnosis. Center – coincidences between main diagnoses (rows) and accessory coma-explaining pathologies detected during ED management (columns), given in absolute numbers. (As we gave priority to diagnoses from class I by definition, only pathologies from classes II and III appear in columns)

### Persistent CUE – multiple pathologies (Table 1 and Additional file 1, Fig. 2a/b)

**Fig. 2:**
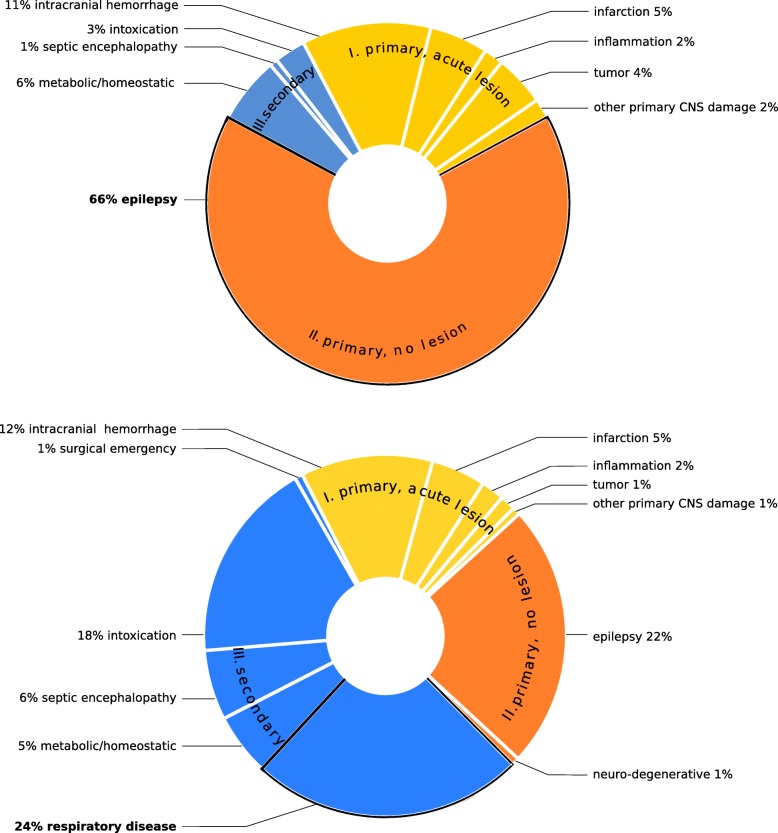
**a** Distribution of main diagnoses in 286 out of 854 patients with persistent CUE either caused by epilepsy or accompanied by epileptic seizures. **b** Distribution of main diagnoses in 144 out of 854 patients with persistent CUE and proof of respiratory insufficiency (CO_2_-narcosis)

Two hundred eighty-two of 854 patients with persistent CUE (33%) presented with more than one CUE-explaining pathology. Accessory pathologies to main diagnoses included acute symptomatic epileptic seizures, medical disorders, intoxication and one surgical emergency. However, theses accessory pathologies were main diagnoses in other patients. For example: epileptic seizures occurred in 286 patients of whom only 188 had epilepsy; severe respiratory insufficiency was present in 144 patients of whom only 35 suffered from a primary respiratory disorder (Fig. 2a/b). There were few instances of concurrent multiple class III pathologies.

### Persistent CUE – in-hospital mortality (Table 2)

In-hospital mortality of patients presenting with persistent CUE was 25% (217/854 pat.; Table 2). In-hospital mortality rates for main diagnoses were 49% (class I), 6% (class II) and 13% (class III; χ^2^-test: *p* < .000). Given the high incidence of accessory pathologies, we compared the in-hospital mortality rate associated with a main diagnosis to the in-hospital mortality rate associated with the same pathology when detected *as an accessory to* another main diagnosis. Again, to highlight the two most striking examples: the in-hospital mortality rate of CUE *caused by epilepsy* is 6% whereas that of CUE *accompanied by epileptic seizures* is more than double (13%). Accordingly, the in-hospital mortality rate of CUE *caused by* septic encephalopathy is 20% whereas the one of CUE *accompanied by* septic encephalopathy is also nearly twice the number (39%). However, the presence of more than one coma-explaining pathology was not associated with a significantly higher in-hospital mortality rate (25% in 572 patients with one pathology vs. 27% in 282 patients with multiple pathologies; χ^2^-test: *p* = .493).

**Table 2:** Numbers of patients who died in-hospital (n/n^†^), ordered by classes of main diagnoses; in-hospital mortality rates (MR) for class of diagnoses. Left – persistent CUE (*n* = 854, 463 males, 391 females; median age 65; median GCS 5). Right – transient CUE (*n* = 173, 107 males, 66 females; median age 65; median GCS [on-site] 8; median GCS [in ED] 14)

Main diagnosis		Persistent CUE			Transient CUE		
		n	n†	MR	n	n†	MR
I	intracranial hemorrhage	190	106	49%	27	6	14%
	infarction	95	47		9	0	
	inflammation	22	5		2	0	
	tumor	17	4		2	0	
	other primary CNS damage	6	1		2	0	
II	epilepsy	188	11	6%	47	1	2%
	neuro-degenerative disease	4	2		1	0	
	psychiatric disease	21	0		13	0	
III	cardiac/pulmonary	53	15	13%	10	1	3%
	metabolic/homeostatic	50	12		11	0	
	septic encephalopathy	25	5		5	0	
	intoxication	165	3		25	0	
	other secondary CNS affection	12	2		17	0	
	surgical emergency	6	4		2	1	

### Transient CUE (Fig. 3, Table 2)

**Fig. 3:**
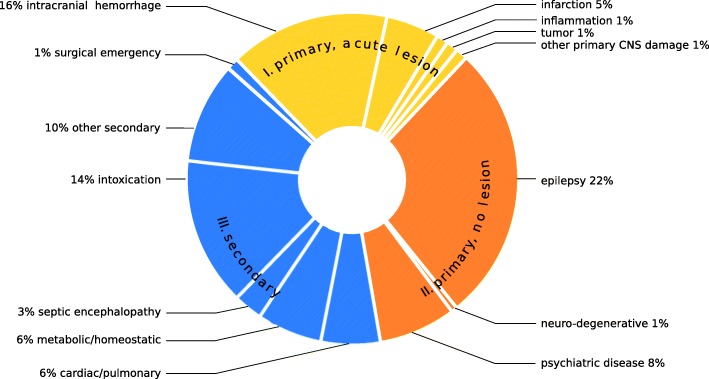
Distribution of main diagnoses in 173 patients with transient CUE (s. Table 2), ordered by classes I-III (107 males, 66 females; median age 65; median GCS [on-site] 8; median GCS [in ED] 14)

Forty-two of 173 transient CUE patients (24%) were diagnosed with a primary CNS pathology with acute structural brain damage (Table 2, Fig. 3). In more than one third (*n* = 16) of these, the transient loss of consciousness could be attributed to acute symptomatic seizures. Main diagnoses from class II were found in 61 transient CUE patients (35%). The largest sub-division of transient CUE patients had main diagnoses from class III (70/173 pat.; 41%). The overall in-hospital mortality rate of patients presenting with transient CUE was substantially lower than in patients with persistent CUE, yet still surprisingly high (5%).

## Discussion

This study reports a large cohort of consecutive ED patients presenting with CUE. The rate of CUE among all ED patients was 0.4% in our study and lower than in previous studies [15, 26]. Two factors may explain this difference. First, we excluded patients with TBI or primary cardiac arrest. Second, there are growing numbers of patients presenting to EDs who do not require emergency services, thus causing a significant crowding effect [27]. Nonetheless, emergency physicians are facing patients with CUE regularly.

The mortality of our CUE patients significantly exceeds the mortality of multiple trauma, stroke or myocardial infarction [28–30]. An efficient diagnostic workflow for CUE patients should therefore be based on a classification of underlying diagnoses that anticipates differential therapeutic consequences as precisely as possible. We have therefore modified the traditional approach, where underlying pathologies are classified into primary (e.g. neurological) and secondary (e.g. medical) causes of coma [1]. This classification does not fully capture the substantial proportion of patients that suffer from primary CNS-related disorders without acute structural damage such as seizures in epilepsy syndromes. These disorders mostly require a less invasive and often less time-sensitive treatment. This is reflected by a lower mortality of patients in this class of diagnoses. The existence of a newly acquired brain lesion including the early stages of an arterial occlusion or CNS inflammation remains to be the crucial classifier that divides class I from the rest. Class I diagnoses are associated with a high in-hospital mortality of nearly 50% which is why these patients require quick decisions that may lead to neurological, neuro-radiological or neurosurgical interventions. Timing is even more important if these options require an emergency referral to a specialist center. Brain imaging should be mandatory for all CUE patients. CSF testing is of equal importance. However, while a CT scan can be performed straight away, an invasive lumbar puncture may be protracted by coagulation testing and may not be necessary if imaging and other laboratory tests have provided unequivocal results in the meantime. All other pathologies (classes II and III) tend to be more difficult to diagnose as there are no solitary classifiers able to identify them.

There are published studies on comparable real-life cohorts of CUE patients in EDs [9]. However, we believe that one important aspect has not yet been explored sufficiently. In real-life conditions, laboratory and imaging results plus fragments of medical history arrive sequentially in the majority of emergencies. Thus, one cannot necessarily reduce the complexity of the diagnostic problem in a given CUE patient down to one diagnosis or to the first pathology detected. To address this problem, we assessed all possibly coma-explaining pathologies in each patient and found that one third of our cohort presented with more than one pathology. While such coincidences may often seem trivial in retrospect, during the first stages of ED management, not all detectable pathologies appear in an obvious causal connection. Most findings need to be dealt with in a fairly random sequence that is mainly determined by logistical rather than medical factors. Thus, consequences risk to be detected before causes. Moreover, confusing symptoms with diagnoses (e.g. epileptic seizures vs. epilepsy) or accessory pathologies with main diagnoses may result in misjudging the severity of the patient’s condition. As a consequence, it may be suggested that CUE patients should receive brain imaging as well as a complete medical and neurological work-up even when a primary CNS pathology is seemingly evident. Therefore, decision points in clinical pathways for acutely comatose patients that rule out diagnostic procedures should not be set in the early diagnostic process.

Although in-hospital mortality is lower in transient CUE, these patients should still be considered high-risk emergencies. While the spectrum of pathologies is not entirely congruent with persistent CUE, it still includes the same diagnoses. Our data further show that transient CUE does by no means exclude a significant acute structural pathology. Approximately 25% of transient CUE patients had class I diagnoses associated with an in-hospital mortality rate of 14%. Regarding the possible fluctuation of GCS values in those patients, setting a certain GCS cutoff value for the management of patients with CUE seems problematic, since it cannot select low risk patients with sufficient specificity. These patients should therefore be managed as quickly according to the same routines as patients with persistent CUE. This applies particularly to epileptic seizures. In an ED setting, unless there is absolute certainty about a previously established diagnosis of epilepsy, possible causes of acute symptomatic seizures should always be ruled out.

The data from our cohort show that the spectrum of conditions underlying CUE is broad and may include a surprisingly high number of coincidences of multiple coma-explaining pathologies. This finding has not been reported so far. Thus, significant pathologies may be masked by initial findings and only appear at the end of the diagnostic work-up. Furthermore, even transient CUE showed a significant mortality, thus rendering GCS cutoffs for selection of high- and low-risk patients questionable. Taken together, our data advocate for a standardized diagnostic work-up that should be triggered by the emergency symptom CUE and not by any suspected diagnosis [19]. This standardized routine should always be completed - even when initial coma-explaining diagnoses may seem evident.

## Limitations

Patients with primary cardiac arrest or TBI were excluded. Even though these patients might have other pathologies as well, their main diagnosis is immediately evident and there are established guidelines for management of these disorders [31–33]. In some patients, pre-hospital intubation and sedation may have artificially sustained coma. However, as this is hard to judge on admission, exclusion of these patients would have biased an otherwise real-life CUE cohort.

## Supplementary information

**Additional file 1.** Left-hand columns – detailed frequencies of main diagnoses in 854 patients with persistent CUE (463 males, 391 females; median age 65; median GCS 5), ordered by classes I-III. Second column of numbers – numbers of patients diagnosed with accessory pathologies besides their main diagnosis. Center – coincidences between main diagnoses (rows) and detailed accessory coma-explaining pathologies detected during ED management (columns), given in absolute numbers. (As we gave priority to diagnoses from class I by definition, only pathologies from classes II and III appear in columns).

## Data Availability

The datasets used and/or analysed during the current study are available from the corresponding author on reasonable request.

## Abbreviations

CNS: Central nervous system
CSF: Cerebro-spinal fluid
CT: Computed tomography
CUE: Coma of unkown etiology
ED: Emergency department
GCS: Glasgow Coma Scale
ICU: Intensive care unit
LOC: Loss of consciousness
MRI: Magnetic resonance imaging
PRES: Posterior reversible encephalopathy syndrome
TBI: Traumatic brain injury
