# Supplementary material for: Causes of brain dysfunction in acute coma: a cohort study of 1027 patients in the emergency department
Source: Scand J Trauma Resusc Emerg Med. 2019 Nov 7;27:101. doi: 10.1186/s13049-019-0669-4 (PMC6836468; doi:10.1186/s13049-019-0669-4)
Supplement: Supplementary file 1 — Additional file 1. Left-hand columns – detailed frequencies of main diagnoses in 854 patients with persistent CUE (463 males, 391 females; median age 65; median GCS 5), ordered by classes I-III. Second column of numbers – numbers of patients diagnosed with accessory pathologies besides their main diagnosis. Center – coincidences between main diagnoses (rows) and detailed accessory coma-explaining pathologies detected during ED management (columns), given in absolute numbers. (As we gave priority to diagnoses from class I by definition, only pathologies from classes II and III appear in columns). [file 13049_2019_669_MOESM1_ESM.pdf]

	diagnosis		[n] patients with main diagnosis	[n] of whom had multiple pathologies	[n] coincidences with accessory pathologies: II		III																[n] main diagnosis + [n] accessory path.
					epileptic state	epileptic seizures	cardiogenic syncope	cardiogenic shock	respiratory insufficiency	exsiccosis	hepatic encephalopathy	hyperosmolar/ketoacidotic coma	hypoglycemia	hyponatremia	uremia	hypothermia	septic encephalopathy	alcohol intoxication	opiate/opioid (+ other) intox.	benzodiazepine (+ other) intox.	psychiatric medication intox.	aortic dissection/bleeding	
I	**intracranial hemorrhage**	sub-tentorial	21	6	1	3			3	1							1						21
		primarily intraventricular	2	1		1																	2
		supra-tentorial, intracerebral	80	15	1	9			5	1													80
		supra-tentorial, subarachnoid	26	10		4	1	1	4									1					26
		supra-tentorial, subdural	31	12	5	3			3		1							4					31
		supra-tentorial, combined	30	11	2	4			2					2	1			1					30
	**infarction**	basilar artery occlusion	26	3					2	1													26
		sub-tentorial, cerebellar	5	2						1							1		1				5
		supra-tentorial, (bi-)thalamic	6	0																			6
		supra-tentorial, middle cerebral artery	58	29	5	10		1	5	11					1		4					1	58
	**venous occlusion**	sinus thrombosis	1	1		1			1														1
	**PRES**	PRES	5	4	3	1																	5
	**inflammation**	meningo-encephalitis	15	9		3			3	5					2		5						15
		aseptic encephalitis	7	2		2										1							7
	**tumor**	focal edema/occlusive hydrocephalus	5	3	2	1			1														5
		first diagnosis of solid tumor	12	10	7	3			1														12
II	**epilepsy**	epileptic state	94	28				1	22	5				1			5	1					123
		epileptic seizures	94	20				2	12	8				1			3	2		1			163
	**neuro-degenerative disease**	LOC in neuro-degenerative disease	2	0																			2
		akinetic crisis (pseudocoma)	2	1					1														2
	**psychiatric disease**	dissociative state (pseudocoma)	21	0																			21
III	**syncope**	non-cardiogenic syncope	2	0																			2
	**cardiac/pulmonary**	cardiogenic syncope	8	2						1							1	2					9
		cardiogenic shock	10	0																			22
		respiratory insufficiency	35	12				3		9		1	1	1	1		7						144
	**metabolic/homoeostatic**	exsiccosis	10	1											1		1						98
		hemorrhagic shock (GI bleeding)	1	1							1												1
		hepatic encephalopathy	9	6		1			3	2					1		1						14
		hyperosmolar/ketoacidotic coma	6	6		2			1	6					3	3	3						7
		hypoglycemia	10	6		3			3	3	1				1		1						11
		hyponatremia	10	9	1	8			1														15
		uremia	4	2	1	1				2							2						21
		hypothermia	0	0																			11
	**septicemia**	septic encephalopathy	25	25	1	1		1	9	25	2				6	3			1		1		62
	**intoxication**	alcohol only	79	12		1			10	1													90
		opiates/opioids (+ other)	34	17		2			12	3													36
		benzodiazepines (+ other)	17	4		1			2							2							18
		psychiatric medication	13	4		1		2	1	1													14
		other substances	22	5		3			1	1						2	1						22
	**unspecified encephalopathy**	unspecified secondary CNS affection	10	0																			10
	**surgical emergency**	aortic dissection/bleeding	5	2				1	1														6
		mesenteric infarction	1	1						1							1						1
			854	282	29	69	1	12	109	88	5	1	1	5	17	11	37	11	2	1	1	1	
